# Evaluation of the Color Stability of Multilayer Zirconia After Exposure to Staining Solutions and Artificial Aging

**DOI:** 10.3390/dj14020077

**Published:** 2026-02-02

**Authors:** Brunilda Koci, Alba Kamberi, Adora Shpati, Olja Tanellari, Balcos Carina, Adela Alushi

**Affiliations:** 1 Department of Stomatology, Faculty of Dental Sciences, Aldent University, 1005 Tirana, Albania; brunilda.koci@ual.edu.al (B.K.); alba.kamberi@ual.edu.al (A.K.); adora.shpati@ual.edu.al (A.S.); oli_koca@yahoo.com (O.T.); adela.alushi@ual.edu.al (A.A.); 2 Surgery Department, Faculty of Dental Medicine, “Grigore T. Popa” University of Medicine and Pharmacy, 700115 Iasi, Romania; 3 Advanced Technology in Medicine & Dentistry, “Gabriele d’Annunzio” University, 66100 Chieti, Italy

**Keywords:** multilayer zirconia, color stability, staining solutions, artificial aging

## Abstract

**Background/Objectives:** Multilayer zirconia restorations can feature a shade gradient or a strength gradient, with layers differing in color or phase composition within the same material. The aim of this in vitro study was to evaluate the color stability in all layers of multilayer zirconia after exposure to staining solutions and artificial aging. **Methods:** Square-shaped specimens (N = 120) of color A2 were fabricated from 4Y-PSZ and 3Y/4Y-PSZ multilayer zirconia—Katana STML, DD Cube One ML, and Katana YML—and their baseline color values (T0) were measured with a clinical spectrophotometer (VITA Easyshade V). The specimens were randomly divided into four groups (n = 10/gp) and immersed in physiologic solution, 0.2% chlorhexidine gluconate (CHX) mouth rinse, and staining coffee solution. Then, they were measured continuously for 7 (T1), 14 (T2), and 21 days (T3). The last group of specimens underwent accelerated aging in a steam autoclave at 134 °C and 2 bar pressure and measured after 1 (T1), 3 (T2), and 5 h (T3). After the immersion process and artificial aging, discoloration values (ΔE) were calculated using the formula ΔE = [(ΔL*)^2^ + (Δa*)^2^ + (Δb*)^2^]^1/2^ and analyzed with the SPSS v 23.0 software with a *p* value < 0.05. **Results:** All specimens showed significant color differences in the T3 measurements after exposure to coffee and CHX, with the highest ΔE values in the enamel layers. Katana YML showed the most significant differences in ΔE in the cervical layers after exposure to artificial aging. **Conclusions:** Multilayer zirconia exhibited dependent optical changes, with the enamel layers being the most affected after exposure to staining solutions. Gradient pigmentation and differences in phase composition caused differences in color to the multilayer zirconia layers after exposure to staining solutions and artificial aging.

## 1. Introduction

Evolutionary developments in the structure and fabrication of polycrystalline zirconia have been made over the years to balance aesthetics and mechanical properties [1]. Improvements in composition and microstructure are critical factors affecting the properties and performance of the material, leading to the development of different generations of zirconia and various multilayer systems [2].

First-generation zirconia (3YTZP), which are stabilized at room temperature with 3 mol% yttrium oxide and a small amount of alumina (0.25 wt%) in the tetragonal phase of the crystalline structure, were used as a framework veneered with feldspathic porcelain due to limited aesthetic results [3]. The so-called toughened zirconia exhibited high mechanical properties with high fracture toughness due to the transformation toughening mechanism, which inhibited crack propagation due to the transformation of tetragonal crystals into the monoclinic phase (t-m), followed by a 3–5% increase in volume. This transformation has been shown to occur in the interface between the framework and the veneering porcelain and is accompanied by differences in their coefficient of thermal expansion (CTE), which contribute to the clinical complications associated with these restorations, such as chipping and delamination of the veneering porcelain. In addition to chipping problems, first-generation zirconia exhibited limited aesthetic results due to the anisotropic behavior of tetragonal crystals [4,5].

To overcome these clinical complications and improve the optical properties of restorations, second-generation zirconia consisting of full-contour monolithic zirconia were introduced onto the market. This monolithic design had tetragonal zirconia polycrystals stabilized with 3 mol% yttria but an alumina content reduced to less than 0.05 wt%, which modestly improved translucency. However, the associated aesthetic results were limited due to the birefringence of tetragonal zirconia and light scattering effects [6].

The reduction of alumina and exposure of zirconia to the oral environment contributed to another phenomenon known as low-temperature degradation (LTD), characterized by a continuous transformation from tetragonal to monoclinic crystals [7]. This hydrothermal degradation, observed since the first generation and accompanied by alterations in the mechanical and optical properties of second-generation zirconia, has been shown to be almost threefold higher [8].

Later generations of zirconia consisted of tetragonal and cubic phases stabilized at room temperature, achieved by adding 3% to 8% yttria to develop monolithic restorations from partially stabilized zirconia [9]. This increase in yttria content in zirconia contributed to higher translucency and optical properties, but reduced mechanical properties as the cubic phase does not undergo stress-induced transformation [10]. Third-generation zirconia consist of monolithic restorations with a 5% yttria content and 50% cubic phase composition, which led to increased translucency and optical properties but a decrease in flexure strength and fracture toughness, similar to lithium disilicates [11]. To balance the optical and mechanical properties, in fourth-generation zirconia approximately 25% of the cubic phase was stabilized at room temperature with 4% yttrium oxide, enabling monolithic restorations suitable for a wider range of indications. The non-birefringent cubic phase has improved translucency and made restorations less vulnerable to aging since it does not undergo phase transformation [12,13]. Another approach to enhancing the esthetic appearance of monolithic restorations was the development of multilayered zirconia, designed to mimic the gradient shade of natural teeth from the more translucent incisal area to the gingival area with increased chroma and opacity, while retaining the same mechanical features and material limitations [14].

This improvement in color with layers corresponding to enamel and dentin in the block of multilayer zirconia was obtained by pressing differently shaded oxide-doped zirconia layers or by immersing pre-sintered zirconia blocks in coloring liquids, affecting the optical and mechanical properties of multilayer zirconia. Polychromatic and hybrid composition multilayer zirconia with enhanced translucency and strength were subsequently introduced onto the market. These restorations resulted in gradient strength and color within the same material, with layers differing in yttria content and cubic phase, progressively increasing from the gingival to the incisal area (M3Y-5Y, M3Y-4Y) [15,16,17].

It has been shown that the composition of zirconia, sintering temperature and duration, number of firings, and low-temperature degradation also contribute to the optical properties, including color [18]. Color was quantified according to the Commision Internationale de l’Eclairage (CIE) using the CIE L*a*b*color coordinates to measure the color of restorations, with ΔE calculated to determine differences in the perceived color. Several studies have reported ΔE > 3.7 as a threshold value for a clinically distinguishable color change, with an acceptable range from 2.72 to 6.8 [19,20,21,22]. According to various authors, ΔE values beyond the acceptable threshold are considered to indicate an acceptable color discrepancy [23,24].

Different studies have shown that color may also be influenced by extrinsic factors related to exposure to mouth rinses, staining solutions, and artificial aging [25].

However, to our knowledge, research focusing on the effects of these factors on the color stability of multilayer blanks, analyzed layer-by-layer along their cross sections, is limited. These studies did not investigate the effects of extrinsic factors on the optical properties of different layers within the material, which may respond differently. The aim of this in vitro study was to fully understand how these layers of shade-gradient and strength-gradient 4Y-PSZ multilayer zirconia differ after exposure to staining solutions and accelerated artificial aging. The null hypothesis was that, after exposure to different conditions, staining solutions, and artificial aging, there would be no differences in the extent of discoloration among the different layers of the tested materials.

## 2. Materials and Methods

Square-shaped specimens with dimensions of 14 × 14 mm^2^ and 1 mm thickness were fabricated from multilayer zirconia: 4Y-PSZ color-gradient Katana STML (Kuraray Noritake Dental Inc., Tokyo, Japan), DD Cube One ML (Dental Direkt GmbH, Spenge, Germany) and strength gradient KATANA™ YML (Kuraray Noritake Dental Inc., Tokyo, Japan) (Table 1).

**Figure 1:**
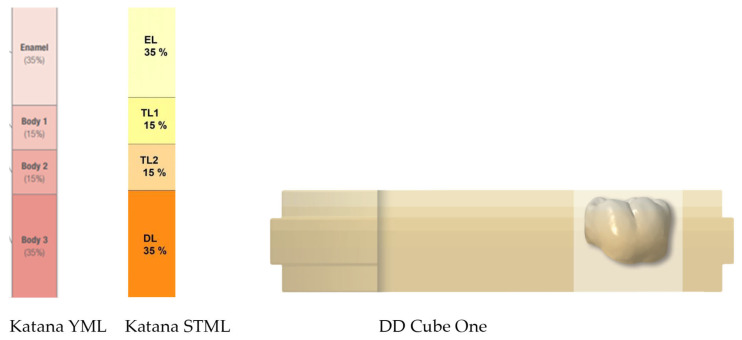
Schematic presentation of the multi-layer zirconia blanks provided by the manufacturers (Kuraray Noritake Dental Inc., Tokyo, Japan and Dental Direkt GmbH, Spenge, Germany).

A standard tessellation language (STL) file was designed with a software program AutoCAD 2020; (AutoCAD 2020; Autodesk Inc., San Rafael, CA, USA) and specimens were milled CORiTEC 150i dry (imes-icore GmbH, Eiterfeld, Germany) from 18 mm zirconia blanks to include all the layers (Figure 1).

Each blank of Katana STML and YML consisted of 4 layers. The nesting position of the specimens was in the center of the blank, resulting in specimens with a 4.3 mm enamel layer, a 5.4 mm transitional layer, and a 4.3 mm body layer. The DD Cube One blank had a fixed 3.5 mm enamel layer, followed by an intermediate layer, and a body layer that increased in thickness as the dimensions of the blank increased. To include all the layers, the nesting position was at the top of the blank for the DD Cube ONE specimens (Table 2).

To standardize the initial color for all specimens, shade A2 was chosen from the VITA shade guide (Vita ZahnFabrik, Bad Säckingen, Waldshut, Germany), and the specimens from preshaded zirconia blanks were sintered in a ceramic furnace (iSINT eco; imes-core GmbH, Eiterfeld, Germany). After sintering, each specimen was sequentially polished using a multistep, diamond-impregnated system (Diacera HP; Eve Ernst Vetter GmbH, Keltern, Germany) under water cooling. Prepolishing, intermediate, and high-gloss steps were performed sequentially at 10,000–8000 rpm with light pressure, followed by a final application of diamond paste (particle size of 1 µm). Plate-shaped, mirror-polished specimens were produced after polishing, providing the most advantageous finish treatment for standardized surface characterization of gradient multilayer zirconia [26,27].

**Figure 2:**
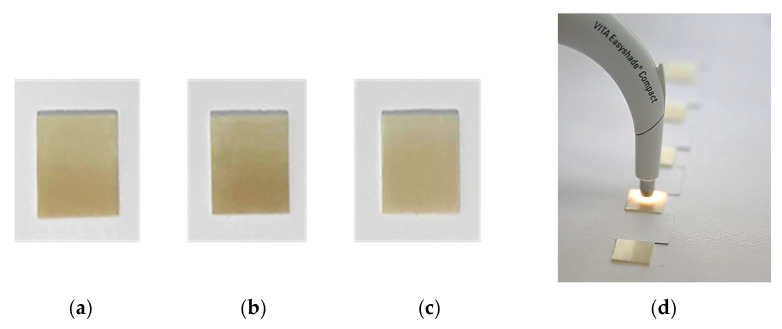
Specimens fabricated from (**a**) Katana STML, (**b**) Katana YML, and (**c**) DD Cube One ML. (**d**) Zirconia specimens and experimental setup used for baseline color measurements.

The thickness of the specimens after polishing was verified using digital calipers (Digimatic Micrometer; Mitutoyo Corp, Kawasaki, Japan). Then, the specimens were ultrasonically cleaned in distilled water for 15 min before testing and individually air-dried for 30 s. Baseline color values (L*, a*, b*) of each specimen were measured (T0) against a white background (CIE L* = 99.07, a* = −0.09, b* = 0.97), as shown in Figure 2, using a reflectance spectrophotometer (VITA Easyshade V, Bad Säckingen, Waldshut, Germany). The device was calibrated before each measurement according to the manufacturer’s instructions, and the white balance was adjusted automatically.

**Figure 3:**
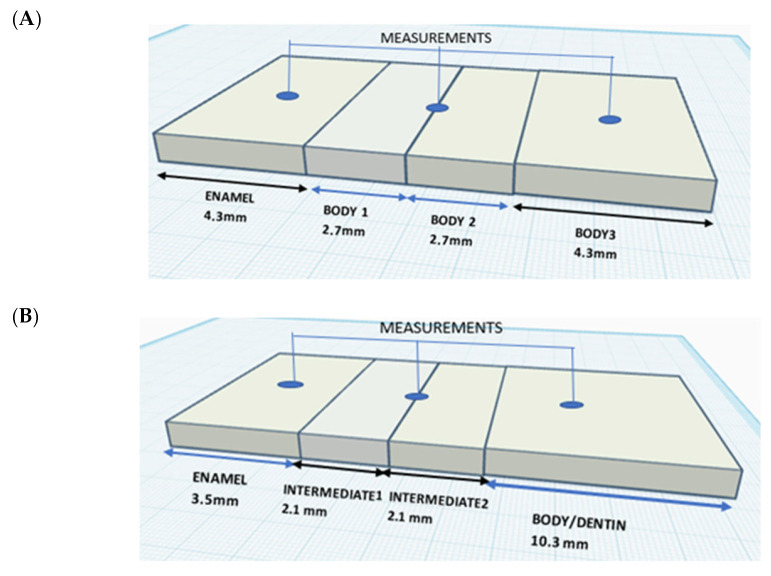
(**A**) Schematic representation of the specimens showing the thickness of individual layers and the relative position of the measurements: (**A**) Katana STML and Katana YML, (**B**) DD Cube One ML.

The color of the specimens was recorded by performing 3 consecutive measurements in each of the layers: enamel, transition/intermediate, and dentine/body layer. A total of 9 measurements for each specimen were conducted, as shown in Figure 3, and the average values of each layer were calculated.

To ensure consistent conditions, the device (with a ø 5 mm aperture) was positioned perpendicular to each specimen under a CIE standard D65 light source. All the measurements were performed by a single calibrated operator to standardize the procedures and to minimize inter-operator variability. The specimens were coded (Z01–Z0120) and randomly allocated within each material to four exposure groups (*n* = 10) by an investigator not involved in the color measurements. The operator performing the color measurements was blinded to the material type and group allocation.

Group 1 specimens were immersed in physiological solution (0.9% sodium chloride, Vioser S.A., Trikala, Greece), whereas groups 2 and 3 were exposed to staining after immersion in 0.2% chlorhexidine gluconate mouthrinse (Curasept S.p.A-Saronno, Italy) and coffee (Landessa Espresso, Ennstal Milch KG, Stainach, Austria), respectively, for a 21-day test period. The staining solutions were changed daily, placed in vials with a cover that prevented their evaporation, and kept in an incubator at 37 °C [28].

Before each measurement, each specimen was rinsed with distilled water for 5 s and gently dried with paper. The measurements were performed after 7 (T1), 14 (T2), and 21 days (T3) of immersion in the solutions. It was found that 24 h of storage time corresponded to 1 month of using the solution [26]. Group 4 underwent an artificial accelerated aging procedure using an autoclave (Kronos B23, Newmedsrl, Reggio Emilia, Italy) at 134 °C and 2 bar pressure for 1 h (T1), 3 h (T2), and 5 h (T3), as recommended in the ISO 13356 standard [29,30].

After the aging protocol, the specimens were dried in air for 24 h. Low-temperature degradation performed in vitro for 1 h in an autoclave was estimated to correspond to 3–4 years in the oral environment [31].

The difference in color (ΔE) for all specimens after the immersion process and artificial aging was calculated by the following formula:

ΔE* = [(ΔL*)^2^ + (Δa*)^2^ + (Δb*)^2^]^1/2^

where ΔL* represents the difference in lightness of the material, and Δa* and Δb* represent the differences in the color coordinates between the exposure times and the baseline.

In the CIELab system, formulas such as ΔEab and ΔE00 are used to determine color differences. Although the ΔE00 formula provides results that better align with the human eye’s perception of color differences, ΔEab is more commonly used in dentistry due to its simplicity and ease of comparison with other studies [32].

Statistical analysis was performed using a statistical software program (IBM SPSS Statistics, v23.0; IBM Corp, Armonk, NY, USA). Descriptive statistics were first calculated to report the mean and standard deviation. The Shapiro–Wilk test was used for assessment of the normality of data. Three-way ANOVA was performed to analyze the effects of different conditions (staining solutions or artificial aging), material brand, and specimen sections on color differences. Tukey’s post hoc tests were used for multiple comparison between groups at a significance level of *p* < 0.05 [33].

## 3. Results

The mean ΔE and standard deviation values of each layer of the specimens were calculated for each measurement time (T1, T2, T3) after immersion in physiological solution, 0.2%, chlorhexidine mouth rinse, coffee, and after performing artificial aging. The results are shown in Table 3.

For each specimen, overall color change is expressed as ΔE (T3 − T1). This derived variable was used as the outcome measure in three-way ANOVA to compare the effects of material type, specimen layer, and staining conditions.

The three-way ANOVA test showed that material type, specimen layers, and exposure to different conditions each significantly influenced ΔE, along with their interactions (*p* < 0.05), except for the interaction of all three factors combined (Table 4).

**Figure 4:**
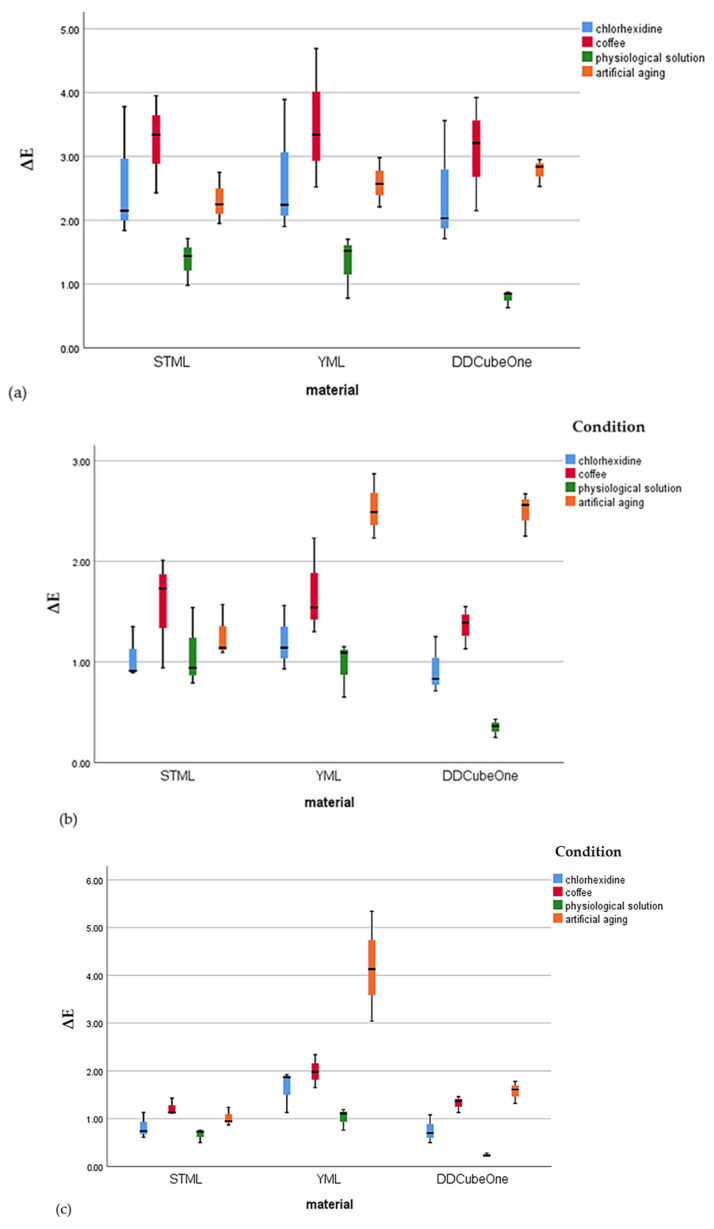
Minimum, maximum, interquartile range, and medians of ΔE for Katana STML, Katana YML, and DD Cube One ML specimens after exposure to four staining conditions (0.2% chlorhexidine mouthrinse, coffee, physiological solution, and artificial aging): (**a**) enamel layer, (**b**) body layer, and (**c**) cervical layer.

Figure 4 illustrates the distribution of ΔE values for the Katana STML, Katana YML, and DD Cube One ML specimens following exposure to the staining conditions (0.2% chlorhexidine mouth rinse, coffee, physiological solution, and artificial aging). Box plots are presented separately for each layer to allow for comparison of the staining response among the materials within each specimen layer.

In the enamel layer, all materials showed the greatest ΔE changes after immersion in coffee and 0.2% chlorhexidine mouth rinse, indicating that this layer was the most susceptible to staining. For the body layer, artificial aging resulted in the highest ΔE values; however, all values remained below the perceptibility threshold, suggesting minimal clinically detectable discoloration. In the cervical layer, Katana YML specimens exhibited the greatest ΔE differences after artificial aging, demonstrating a higher sensitivity of this layer to accelerated aging compared with the other materials.

The Katana STML specimens showed significantly higher ΔE values in the enamel layer compared with the body and cervical layers across all evaluations after exposure to coffee, 0.2% chlorhexidine mouth rinse, and artificial aging, except in the physiological solution group. However the ΔE values remained within clinically acceptable limits for all conditions, except after immersion in coffee and 0.2% chlorhexidine mouth rinse, where the values exceeded the perceptibility threshold, varying from 3.78 to 3.95 (ΔE = 3.7).

The Katana YML specimens exhibited higher ΔE values that exceeded the perceptibility threshold in the enamel layer after immersion in coffee and 0.2% chlorhexidine mouth rinse and in the cervical layer after exposure to artificial aging. After artificial aging, the ΔE values in the cervical layer were statistically significantly higher than those in the enamel and body layers; however, these values remained within the clinically acceptable threshold (ΔE = 6.8).

The DD Cube One ML specimens showed significantly higher color change (ΔE) values in the enamel layer compared with the body and the cervical layers. However, only the ΔE values of the enamel layer after immersion in coffee exceeded the perceptibility threshold of 3.7 (ΔE = 3.92).

## 4. Discussion

The data presented in our study reveal differences in the extent of discoloration among different layers and between both tested materials—shade-gradient and strength-gradient multilayer zirconia—after exposure to staining solutions and artificial aging; therefore, the null hypothesis was rejected.

Different studies have demonstrated that, despite the improved mechanical and aesthetic properties of zirconia, exposure to extrinsic factors (e.g., various beverages and mouth rinses) in the oral environment causes color changes and increases surface roughness. Irregular and rough surfaces induce plaque accumulation and staining, resulting in increased discoloration of zirconia restorations [34,35,36].

The optical behavior of ceramic and surface characteristics influence long-term clinical success of restoration, impacting patient satisfaction [37]. Discoloration has been reported as one of the main reasons for the replacement of a restoration [38]. Color stability was evaluated in all layers of shade- and strength-gradient specimens in our study, with a greater number of measurements per specimen, in contrast to several studies that evaluated each layer separately. The specimens were 1 mm thick and polishing was performed as a finishing laboratory treatment, as suggested by several studies [26,27].

Immersion in coffee resulted in more discoloration than in 0.2% chlorhexidine mouth rinse. This discoloration was clinically distinguishable (ΔE > 3.7) for the enamel layers of all brands of zirconia, with Katana YML exhibiting the highest color change. Discoloration of the enamel layer was statistically higher compared to the body and cervical layers in the specimens of all zirconia brands (*p* < 0.05). The body and cervical layers exhibited better color stability, with decreased ΔE values that were lower than the clinically perceptible level.

These results are in concordance with a previous study that reported zirconia specimens to have perceivable discoloration after immersion in coffee, with a significant increase in ΔE values [39]. The same findings were reported by Haralur et al., with zirconia specimens showing greater discoloration after exposure to coffee, despite using a different methodology with a shorter immersion period and measurements taken after also performing hydrothermal aging [34]. Coffee has a high potential for staining and discoloration due to the presence of tannin and chlorogenic acids. Coffee as a staining solution was identified as the most chromogenic substance, which is in accordance with two other studies [35,36].

The study by Alzahrani et al. also evaluated immersion of zirconia specimens in 0.2% chlorhexidine mouth rinse, and a marked color change was reported in shade-gradient multilayer zirconia of different brands with varying yttria concentrations. In our study, immersion in 0.2% chlorhexidine mouth rinse resulted in color changes that exceeded the perceptibility threshold (ΔE = 3.7) for enamel layers of Katana STML and YML. This result was statistically significant (*p* < 0.05) compared to other layers of these zirconia specimens [35].

Shade-gradient zirconia of DD Cube One ML specimens exhibited significant increases in ΔE values for the enamel layers compared to the body and cervical layers, but these color changes were below the perceptibility threshold. Different pigmentations of zirconia brands influence the color stability of zirconia specimens [40].

The stainability of teeth and restorative materials by 0.2% chlorhexidine mouth rinse has been described as a major drawback. The discoloration mechanism involves the interaction of chromogens with polyvalent metal salts (e.g., iron supplements) and cationic antiseptics (e.g., chlorhexidine gluconate) which are often colorless [41]. The deterioration of chlorhexidine molecules releases parachloranilin, resulting in a denaturing protein and the formation of metal sulfides [42]. The potential of 0.2% chlorhexidine gluconate mouth rinse to cause staining of zirconia has also been confirmed by other studies [43]. Alpkilic et al. investigated the color stainability of different ceramic materials and reported that 0.2% chlorhexidine mouth rinse induced higher ΔE values among all mouth rinses, also recommending its use with caution [28]. In comparison with the findings of Sayed et al., both studies reported color alteration after exposure to 0.2% chlorhexidine mouth rinse; however, the distribution of ΔE across layers in our research reveals novel insight into the differential behaviors of strength- and shade-gradient multilayer zirconia [44].

Exposure to staining solutions, coffee, and 0.2% chlorhexidine mouth rinse resulted in more discoloration in the enamel layer compared to the body and cervical layers of both the shade- and strength-gradient multilayer zirconia. As described by Kolakarnprasert et al., shade-gradient multilayer zirconia exhibited different pigment concentrations within different layers, with the lowest percentage of metal oxides in the enamel layer and increasing chroma toward the cervical layers. This contributes to enamel layers being less saturated, with higher L*values resulting in more perceivable color changes after exposure to staining from extrinsic pigments. An analysis of different layers of strength-gradient multilayer zirconia by Ikonoshi et al. suggested that the enamel layers exhibited a different yttria content compared to the body layers, resulting in different crystalline structures with different translucency [25].

Higher translucency of the enamel layer, due to the higher yttria content and the cubic phase, allows for greater light transmission with less internal scattering, making staining from chromogenic substances produce a greater perceived color difference (ΔE). These differences in both shade- and strength-gradient zirconia make the enamel layer less resistant to staining, exhibiting higher discoloration compared to the body and cervical layers.

The null hypothesis—which stated that there would be no difference in the extent of discoloration among different layers of multilayer zirconia after accelerated aging—was rejected as differences in the color of shade-gradient and strength-gradient zirconia layers were observed.

Strength-gradient zirconia (Katana YML) exhibited color changes in the cervical layer (body 3 layer), exceeding the clinical perceptibility threshold (ΔE > 3.7), which was statistically significant compared to the discoloration observed in the other layers of these specimens. This color change was influenced by the aging behavior of the strength-gradient body 3 layer, which strongly depends on the tetragonal phase crystalline structure stabilized with 3 mol% yttrium oxide [45].

Differences in pigmentation between layers of strength-gradient zirconia also contributed to differences in aging rates within the specimens, with layers containing pigment agglomerates showing greater transformation and high aging rates. The exact mechanism of color instability is not very clear, but different studies have suggested that thermal conditions may influence coloring pigments, causing pigments to break down and resulting in changes in lightness and blue-yellow coordinates [46,47].

In the present study, differences in the color parameters of shade-gradient zirconia layers were observed after accelerated artificial aging, but these differences were below the clinical perceptibility threshold (ΔE < 3.7) in all measurements. These results are different from the findings of Daoud MZ et al., who reported uniformly shade stability across various CAD/CAM ceramics after hydrothermal aging, whereas our findings show that multilayer zirconia exhibited layer-dependent color changes [48]. Our findings are similar to those of Miura et al., who evaluated color differences before and after artificial aging of shade-gradient and strength-gradient zirconia and reported greater ΔE values in zirconia with multiple compositions [45]. Different studies have suggested that hydrothermal aging changes the optical properties of zirconia, with degradation beginning on the surface and higher surface degradation found in conventional zirconia rather than in highly translucent zirconia [49,50].

Our findings demonstrate that color stability is not determined by the material alone, but by the interaction between different crystalline compositions and the specific staining environment. Strength-gradient zirconia exhibited greater color shifts than shade-gradient zirconia when exposed to coffee and 0.2% chlorhexidine mouth rinse, particularly in the enamel layer. Additionally, significantly greater ΔE values were observed in the cervical layers after artificial aging, likely due to the crystalline structure of the body 3 layer. These results indicate that multilayer zirconia do not behave uniformly from an optical view; instead, their layer-specific composition interacts with the staining environment. Clinically, this suggests that mechanical performance and color stability should be balanced based on the anticipated chromogenic agents when selecting a material: shade-gradient multilayer zirconia are preferred in highly aesthetic zones or in patients with high exposure to staining conditions, while strength-gradient multilayer zirconia are preferable in clinical situations where functional demands are prioritized. Overall, these findings have direct implications for selecting the appropriate material to achieve longer lasting optical outcomes with minimal need for maintenance over time.

A limitations of the present study is that the zirconia specimens were not analyzed for surface texture, topography, or roughness after exposure to different conditions. Additionally, only one shade of both the shade- and strength-gradient multilayer zirconia was studied. Finally, there was a lack of in vivo conditions of tooth brushing after exposure to staining and artificial aging. Clinical studies are necessary to evaluate the optical properties of shade-gradient and strength-gradient zirconia after exposure to different conditions.

## 5. Conclusions

Based on the results of this in vitro study, the following conclusions were drawn: the color of the shade-gradient and strength-gradient zirconia layers was influenced by exposure to staining solutions and artificial aging. Both the shade-gradient and strength-gradient zirconia exhibited more pronounced discoloration in the enamel layers after immersion in coffee and 0.2% chlorhexidine mouth rinse. In addition, the strength-gradient zirconia showed significantly higher ΔE values in the cervical layers after artificial aging, which can be attributed to differences in phase composition between these materials.

## Figures and Tables

**Table 1:** Tested multilayered zirconia.

Material	Manufacturer	Composition	Lot Nr
Katana STML: 4Y PSZ, Multilayered	Kuraray Noritake Dental Inc., Tokyo, Japan	87–92% ZrO_2_ + HfO_2_ 7–9% Y_2_O_3_; 0–2% other oxides	EH0TK
Katana YML: 3–4Y PSZ, Multilayered	Kuraray Noritake Dental Inc., Tokyo, Japan	ZrO_2_: 87–92%; Y_2_O_3_: 8–11; HfO_2_: ≤5.0; Al_2_O_3_: ≤1.0; Other oxides: ≤1.5	E0LWV
DD Cube One ML: 4Y TZP, Multilayered	Dental Direkt GmbH, Spenge, Germany	ZrO_2_ + HfO_2_ + Y_2_O_3_ ≥ 99.0; Y_2_O_3_ < 8; Al_2_O_3_ < 0.15; Other oxides <1.0	7162424019

The minimum total sample size required was 120 specimens (10 per group for 12 groups), as determined by statistical power analyses using G Power Program (G * Power 3.1, Heinrich Heine University, Düsseldorf, Germany) based on a sensitivity of 0.4 with a margin error of 5% and power of 80%.

**Table 2:** Dimensions of each layer of zirconia blanks.

Material	Blank (Height, mm)	Layer 1 (mm/%)	Layer 2 (mm/%)	Layer 3 (mm/%)	Layer 4 (mm/%)
Katana YML	18	Enamel 6.3/35	Body 1 2.7/15	Body 2 2.7/15	Body 3 6.3/35
Katana STML	18	Enamel 6.3/35	Transition Layer 1 2.7/15	Transition Layer 2 2.7/15	Dentine 6.3/35
DD Cube One ML	18	Incisal 3.5/19.4	Intermediate 2.1/11.6	Intermediate 2.1/11.6	Body/Dentine 10.3/57.2

**Table 3:** Mean ΔE ± SD values of discoloration after exposure of the specimens to staining solutions and artificial aging.

Material	Time	Layer	Condition			
			Physiological Solution	Chlorhexidine 0.2%	Coffee	Artificial Aging
			*Mean + STD*	*Mean + STD*	*Mean + STD*	*Mean + STD*
**Katana STML**	T1	Enamel	0.98 ± 0.37	1.84 ± 0.63	2.43 ± 0.56	1.95 ± 0.23
	T1	Body	0.79 ± 0.55	0.89 ± 0.66	0.94 ± 0.44	1.09 ± 0.35
	T1	Cervical	0.50 ± 0.76	0.61 ± 0.78	1.12 ± 0.76	0.87 ± 0.73
	T2	Enamel	1.44 ± 0.32	2.15 ± 0.19	3.34 ± 0.69	2.25 ± 0.45
	T2	Body	0.94 ± 0.57	0.91 ± 0.63	1.73 ± 0.27	1.14 ± 0.75
	T2	Cervical	0.73 ± 0.85	0.74 ± 0.58	1.13 ± 0.76	0.95 ± 0.13
	T3	Enamel	1.71 ± 0.33	3.78 ± 0.18	3.95 ± 0.84	2.75 ± 0.44
	T3	Body	1.54 ± 0.42	1.35 ± 0.51	2.01 ± 0.29	1.57 ± 0.39
	T3	Cervical	0.76 ± 0.31	1.13 ± 0.79	1.43 ± 0.28	1.24 ± 0.61
**Katana YML**	T1	Enamel	0.78 ± 0.37	1.90 ± 0.76	2.52 ± 0.88	2.21 ± 0.37
	T1	Body	0.65 ± 0.74	0.93 ± 0.45	1.30 ± 0.34	2.23 ± 0.19
	T1	Cervical	0.76 ± 0.65	1.13 ± 0.46	1.65 ± 0.57	3.04 ± 0.34
	T2	Enamel	1.52 ± 0.25	2.24 ± 0.68	3.34 ± 0.42	2.57 ± 0.58
	T2	Body	1.09 ± 0.32	1.14 ± 0.48	1.54 ± 0.71	2.49 ± 0.53
	T2	Cervical	1.11 ± 0.37	1.87 ± 0.75	1.98 ± 0.22	4.13 ± 0.71
	T3	Enamel	1.70 ± 0.29	3.89 ± 0.73	4.69 ± 0.68	2.98 ± 0.78
	T3	Body	1.15 ± 0.48	1.56 ± 0.31	2.23 ± 0.54	2.87 ± 0.44
	T3	Cervical	1.19 ± 0.52	1.92 ± 0.65	2.34 ± 0.32	5.34 ± 0.52
**DD Cube ONE ML**	T1	Enamel	0.63 ± 0.53	1.71 ± 0.12	2.15 ± 0.52	2.53 ± 0.24
	T1	Body	0.25 ± 0.78	0.71 ± 0.95	1.13 ± 0.43	2.25 ± 0.32
	T1	Cervical	0.23 ± 0.27	0.50 ± 0.78	1.13 ± 0.89	1.32 ± 0.37
	T2	Enamel	0.85 ± 0.23	2.03 ± 0.15	3.21 ± 0.24	2.84 ± 0.34
	T2	Body	0.36 ± 0.25	0.83 ± 0.22	1.39 ± 0.39	2.56 ± 0.28
	T2	Cervical	0.23 ± 0.02	0.70 ± 0.30	1.37 ± 0.29	1.61 ± 0.42
	T3	Enamel	0.87 ± 0.22	3.56 ± 0.28	3.92 ± 0.29	2.95 ± 0.74
	T3	Body	0.43 ± 0.10	1.25 ± 0.42	1.55 ± 0.41	2.67 ± 0.31
	T3	Cervical	0.28 ± 0.34	1.08 ± 0.79	1.46 ± 0.41	1.78 ± 0.39

Katana STML layers: enamel, body (transition layers 1 and 2), and cervical (dentine); Katana YML layers: enamel, body (body 1 and body 2), and cervical (body 3); DD Cube One ML layers: enamel (incisal layer), body (intermediate 1 and intermediate 2), cervical (body/dentine).

**Table 4:** Three-way ANOVA results for ΔE.

Source	Type III Sum of Squares	Df	Mean Square	F	Sig.
Corrected model	93.799	35	2.680	9.796	0.000 *
Intercept	316.830	1	316.830	1158.117	0.000 *
Material	8.546	2	4.273	15.619	0.000 *
Layer	25.011	2	12.505	45.711	0.000 *
Solution	33.446	3	11.149	40.752	0.000 *
Material * layer	5.297	4	1.324	4.841	0.002 *
Material * solution	6.946	6	1.158	4.232	0.001 *
Layer * solution	9.298	6	1.550	5.664	0.000 *
Material * layer * solution	5.255	12	0.438	1.601	0.111
Error	19.697	72	0.274		
Total	430.326	108			
Corrected total	113.496	107			

* Differences are statistically significant at *p* < 0.05.

## Data Availability

The original contributions presented in this study are included in the article. Further inquiries can be directed to the corresponding author.
